# Transphosphorylation of *E. coli* Proteins during Production of Recombinant Protein Kinases Provides a Robust System to Characterize Kinase Specificity

**DOI:** 10.3389/fpls.2012.00262

**Published:** 2012-11-30

**Authors:** Xia Wu, Man-Ho Oh, Hyoung Seok Kim, Daniel Schwartz, Brian S. Imai, Peter M. Yau, Steven D. Clouse, Steven C. Huber

**Affiliations:** ^1^Department of Plant Biology, University of IllinoisUrbana, IL, USA; ^2^Department of Physiology and Neurobiology, University of ConnecticutStorrs, CT, USA; ^3^Protein Sciences Facility, Carver Biotechnology Center, University of IllinoisUrbana, IL, USA; ^4^Department of Horticultural Science, North Carolina State UniversityRaleigh, NC, USA; ^5^Agricultural Research Service, United States Department of AgricultureUrbana, IL, USA

**Keywords:** BRI1, BAK1, PEPR1, FLS2, CDPK, phosphorylation motif

## Abstract

Protein kinase specificity is of fundamental importance to pathway regulation and signal transduction. Here, we report a convenient system to monitor the activity and specificity of recombinant protein kinases expressed in *E. coli*. We apply this to the study of the cytoplasmic domain of the plant receptor kinase BRASSINOSTEROID-INSENSITIVE 1 (BRI1), which functions in brassinosteroid (BR) signaling. Recombinant BRI1 is catalytically active and both autophosphorylates and transphosphorylates *E. coli* proteins *in situ*. Using enrichment approaches followed by LC-MS/MS, phosphosites were identified allowing motifs associated with auto- and transphosphorylation to be characterized. Four lines of evidence suggest that transphosphorylation of *E. coli* proteins by BRI1 is specific and therefore provides meaningful results: (1) phosphorylation is not correlated with bacterial protein abundance; (2) phosphosite stoichiometry, estimated by spectral counting, is also not related to protein abundance; (3) a transphosphorylation motif emerged with strong preference for basic residues both N- and C-terminal to the phosphosites; and (4) other protein kinases (BAK1, PEPR1, FLS2, and CDPKβ) phosphorylated a distinct set of *E. coli* proteins and phosphosites. The *E. coli* transphosphorylation assay can be applied broadly to protein kinases and provides a convenient and powerful system to elucidate kinase specificity.

## Introduction

Protein phosphorylation on serine, threonine, and tyrosine residues is catalyzed by protein kinases that transfer the phosphate moiety from ATP to the modified residues (Hanks and Hunter, 1995). In humans, there are more than 500 kinases (Manning et al., 2002) that phosphorylate thousands of identified human phosphosites (Hornbeck et al., 2012). Such a large-scale phosphorylation network is also present in plants. In *Arabidopsis*, a model plant with a relatively small genome, almost 1000 protein kinases have been identified (Chevalier and Walker, 2005). The number of kinases is expected to be higher in crop plants as most of them have a much larger genome (Feuillet et al., 2011). Receptor-like kinases (RLKs) comprise the largest kinase family in the plant kinome; there are more than 600 in *Arabidopsis*, and more than 1200 RLKs in rice (Shiu et al., 2004). To elucidate the complex phosphorylation regulatory networks in eukaryotes, we need to better understand the specificity of the kinases. Kinase specificity involves recognition of short amino acid sequences surrounding the phosphorylated residue, commonly referred to as the phosphorylation motif, and other factors such as secondary and tertiary structure of the site, docking sites on the substrate protein, and co-localization of kinase and substrate (Kennelly and Krebs, 1991; Newton, 2001; Fujii et al., 2004). In plants and animals, advances in mass spectrometry have allowed identification of numerous phosphosites, but in most cases it is not clear which kinase(s) is responsible for the phosphorylation. Hence, analysis of kinase specificity remains an important area of study.

The conventional approach to characterize kinase specificity is through *in vitro* kinase phosphorylation assays on peptides or proteins (Jia et al., 2008), where each substrate-kinase pair is assayed for activity. The use of protein chips and peptide libraries in the assay further enhances the capacity of the screening (Zhu et al., 2000; Mok et al., 2010). However, such assays occur in isolated systems, require laborious kinase purification, and the scope is necessarily restricted to the particular peptides or proteins tested. An alternative approach to characterize kinase specificity is to couple chemical cross-linking technology with engineered kinases that can utilize ATP analogs, such that the proteins labeled can be cataloged as specific substrates for the engineered kinases. Such a method has successfully identified novel substrates for ERK2, Src, and CDKs (Eblen et al., 2003; Ubersax et al., 2003; Ulrich et al., 2003). Unfortunately, not all kinases can be manipulated to the extent necessary for the chemical cross-linking and hence has limited application.

In our research, we are particularly interested in the receptor kinases involved in brassinosteroid (BR) signaling. BRASSINOSTEROID-INSENSITIVE 1 (BRI1) is one of the best characterized receptor kinases in plants and functions with BRI1-ASSOCIATED RECEPTOR KINASE 1 (BAK1) to trigger the intracellular signaling cascades that control plant development and stress responses (Clouse et al., 1996; Li and Chory, 1997). Several downstream BRI1 transphosphorylation substrates have been identified including BKI1, BSK1, and eIF3/TRIP-1 (Ehsan et al., 2005; Tang et al., 2008; Wang et al., 2008; Jaillais et al., 2011), but in general the kinase specificity of BRI1 is not well understood. We recently reported (Oh et al., 2012) that numerous *E. coli* proteins were phosphorylated on tyrosine, threonine, and serine residues during production of the cytoplasmic domain of BRI1, expressed as an N-terminal Flag-tag fusion protein (hereafter referred to as Flag-BRI1). However, while specific sites on bacterial proteins were phosphorylated, it was not clear that the transphosphorylation activity observed was not simply opportunistic phosphorylation of abundant bacterial proteins. In the present study, we wanted to further validate this system. To do this, we further characterized the *E. coli* proteins phosphorylated by Flag-BRI1, and then compared the results with the proteins and sites phosphorylated during expression of four other plant protein kinases. Three of the protein kinases tested are receptor kinases: BAK1, PEP1 RECEPTOR (PEPR1), and FLAGELLIN-SENSING 2 (FLS2). BAK1, as noted above, is co-receptor with BRI1 in BR signaling (Li et al., 2002), and also with FLS2 for microbe-associated molecular patterns sensing (Chinchilla et al., 2007), and PEPR1 for the damage-associated molecular patterns sensing (Krol et al., 2010). The autophosphorylation of BAK1 has been extensively characterized (Wang et al., 2008; Karlova et al., 2009; Oh et al., 2010), and while a few transphosphorylation substrates of BAK1 have been reported, such as BRI1, BIK1, and PUB12/13 (Wang et al., 2005; Lu et al., 2010, 2011), the identity of specific transphosphorylation sites is generally limited. The other two receptor kinases included in this study, FLS2 and PEPR1, are less well characterized in terms of their kinase specificities. In particular, FLS2, which is a non-RD-type protein kinase (Dardick and Ronald, 2006), is considered to have lower kinase activity than BRI1, BAK1, and PEPR1, which are RD-type kinases (Johnson et al., 1996). The fourth kinase tested was soybean calcium-dependent protein kinase (CDPK) β. The CDPKs are soluble kinases, and are important signaling elements for plant stress responses (Cheng et al., 2002). CDPKs can directly bind Ca^2+^, which releases the autoinhibitory domain from the kinase domain, thereby stimulating kinase activity (Harper et al., 2004). Comparative studies with the five protein kinases identified many new sites of autophosphorylation and also allowed us to analyze motifs associated with transphosphorylation catalyzed by each kinase. The results suggest that phosphorylation of *E. coli* proteins during production of recombinant protein kinases provides meaningful insights into the intrinsic specificity of the kinase and may have utility in the characterization of protein kinases from diverse organisms.

## Materials and Methods

### Materials

The genes mentioned in this study are BRI1 (At4g39400), mBRI1 (At4g39400 with K911E mutation), BAK1 (At4g33430), FLS2 (At5g46330), PEPR1 (At1g73080), CDPKβ (O24430_soybean), and 14−3−3ω (At1g78300). The cytoplasmic domains of the receptor kinases were cloned in the pFlag-Mac vector (Sigma–Aldrich, St. Louis, MO, USA), the full-length protein of 14−3−3ω was cloned in the pET-15b vector (Novagen, EMD Millipore, Billerica, MA, USA), and GmCDPKβ was cloned in the pRSET vector (Invitrogen, Carlsbad, CA, USA). Vectors containing genes of interest were introduced to *E. coli* BL21 (DE3) through plasmid transformation. *E. coli* cells were grown in LB medium. For CDPKβ, additional 1 mM Ca^2+^ was added to the growth medium to enhance the activation of CDPKβ. The expression of kinases or proteins was induced with 0.3 mM IPTG when the OD_600_ of *E. coli* cells reached 0.6. After IPTG induction, *E. coli* cells were incubated at room temperature with shaking for the indicated time (up to 16 h).

### Phosphoprotein analysis and mass spectrometry

*E. coli* cells were harvested by centrifugation and resuspended in a buffer containing 50 mM MOPS (pH 7.5) and 150 mM NaCl, before being lysed by sonication. Cell lysates were fractionated by centrifugation at 35,000 × *g* into soluble and pellet (referred to as “p-bodies”) fractions. Recombinant protein kinases in the soluble fractions were removed by incubation with either Flag- or His-affinity beads as appropriate, in order to improve the coverage of the endogenous *E. coli* proteins. Proteins in the soluble fraction were analyzed by SDS–PAGE and immunoblotting with anti-phosphothreonine antibodies (catalog number 71-8200, Invitrogen, Carlsbad, CA, USA) or staining with ProQ Diamond Phosphoprotein Stain (Invitrogen) to monitor overall phosphorylation of bacterial proteins (as in the experiment presented in Figure 4). For mass spectrometric analysis, proteins in the soluble fraction were precipitated with 80% acetone at −20°C overnight, and the resulting pellet was resuspended in buffer containing 6 M urea and 50 mM NH_4_HCO_3_. Proteins in the p-bodies (original extract pellet) fractions were directly extracted with 6 M urea and 50 mM NH_4_HCO_3_. Protein concentration was measured with the Bradford assay (Bio-Rad, Hercules, CA, USA) and 5 mg of total soluble protein or 2.5 mg protein from the pellet fraction was subjected to trypsin digestion as previously described (Wu et al., 2011). The tryptic peptides were collected with C18 SPE columns (Discovery Sciences, Deerfield, IL, USA) and dried. For phosphopeptide enrichment using a TiO_2_ column (Pierce, Thermo Fisher Scientific, Rockford, IL, USA), peptides were dissolved in a buffer containing 25% lactic acid, 0.3% trifluoroacetic acid, and 55% acetonitrile, pH 3.0. The enrichment was processed following manufacturer’s instructions and the phosphopeptides were eluted with 5% NH_4_OH (pH 10.0), and subsequently with 5% pyrolidine (pH 11.0). The eluted peptides were immediately acidified with 5% trifluoroacetic acid to pH < 3.0. For the phosphopeptide enrichment using IMAC (Fe^2+^; Sigma–Aldrich), peptides were dissolved in a buffer containing 250 mM acetic acid, and 30% acetonitrile, pH 3.0, and incubated with the IMAC beads for 30 min at room temperature with shaking. The bound peptides were eluted with 400 mM NH_4_OH (pH 10.0) and acidified with 5% trifluoroacetic acid to pH < 3.0. The eluted peptides from both methods were cleaned up with graphite spin columns (Pierce) before mass spectrometry analysis.

**Figure 1:**
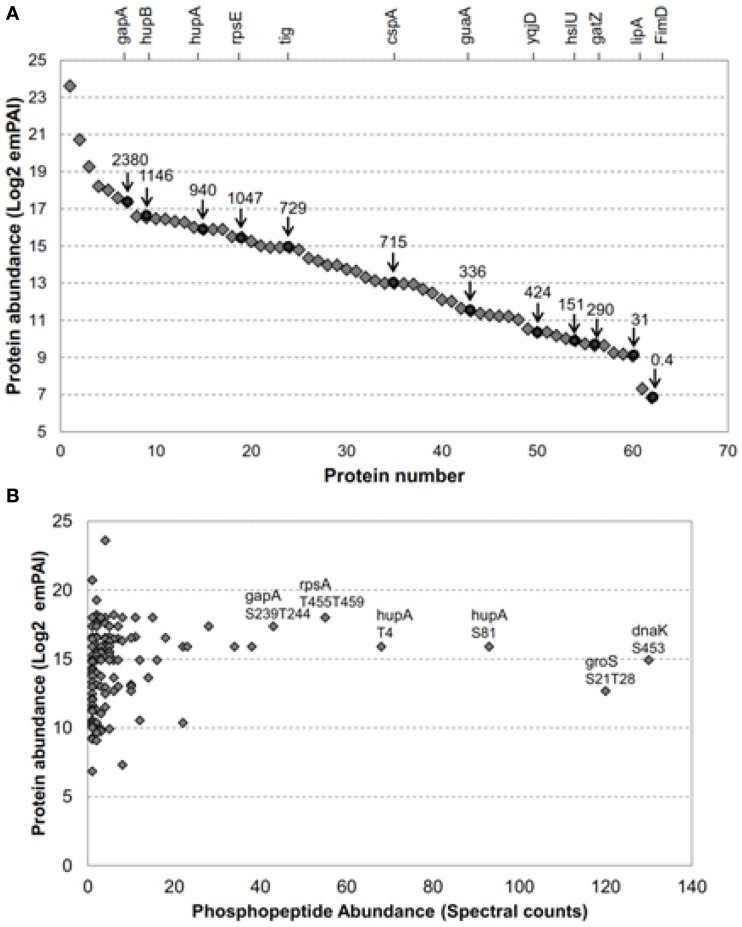
**The *E. coli* proteins transphosphorylated by BRI1 covered a broad range in protein abundance**. **(A)** Relative abundance of 62 of the 73 *E. coli* proteins transphosphorylated by BRI1 *in situ*. Blue diamonds are emPAI-derived copy number per cell (Ishihama et al., 2008) and red circles are values based on fluorescence measurements (Taniguchi et al., 2010) and the corresponding protein names are indicated on the top of the figure. The identity of the proteins quantified by emPAI method were: (1) rplV; (2) rpmD; (3) rplX; (4) crr; (5) rpsA; (6) ahpC; (7) gapA; (8) rpsB; (9) hupB; (10) rpsD; (11) fugA; (12) rpsC; (13) rplC; (14) tsf; (15) rplM; (16) hupA; (17) rpsL; (18) rpmE; (19) rpsE; (20) rplO; (21) rplE; (22) rpsM; (23) tig; (24) dnaK; (25) rpsF; (26) rplN; (27) rplJ; (28) grpE; (29) rpmA; (30) frr; (31) rpsJ; (32) rplK; (33) rpsl; (34) cspA; (35) rpsK; (36) sucB; (37) rpoA; (38) groS; (39) pnp; (40) rho; (41) rpoC; (42) juaA; (43) typA; (44) ybeD; (45) infB; (46) sucD; (47) pta; (48) ihfB; (49) yqjD; (50) rpoD; (51) ihfA; (52) aspA; (53) hslU; (54) ftsZ; (55) gatZ; (56) dnaJ; (57) accD; (58) lipA; (59) gatD; (60) tatA; (61) parB; (62) FimD. **(B)** Abundance of specific phosphopeptides, derived from *E. coli* proteins transphosphorylated by BRI1 *in situ*, was not related to the abundance of the parent protein. The spectral counts were the sum of four independent experiments, two with TiO_2_ enrichment and two with IMAC (Fe^2+^) enrichment. The phosphopeptides with top spectral counts are annotated in the graph.

**Figure 2:**
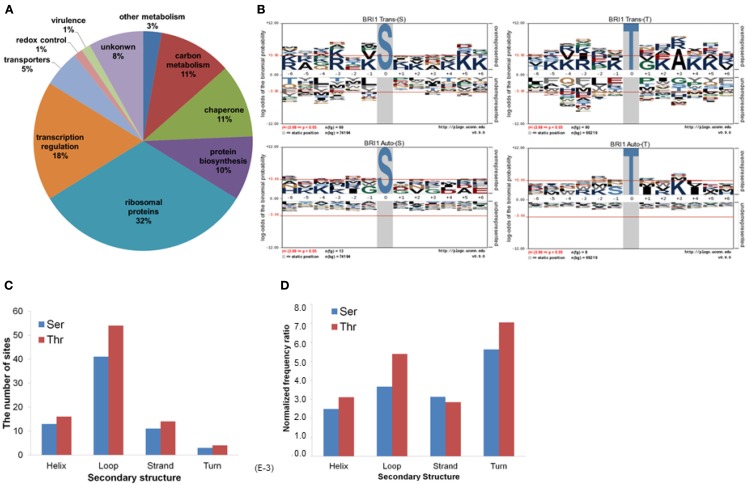
**Characterization of BRI1 transphosphorylationsubstrates in *E. coli***. **(A)** The *E. coli* proteins phosphorylated by BRI1 *in situ* function in diverse biological pathways. **(B)** pLogo motif analysis of BRI1 transphosphorylation (upper panel) and autophosphorylation (lower panel) sites in *E. coli* separated according to serine and threonine sites. The number of sites analyzed is indicated in each panel. The phosphorylated residue is annotated as position 0, and the six upstream or downstream residues are annotated as −6 to −1 and +1 to +6, respectively. Residues above the *x*-axis are overrepresented, relative to their statistical significance in the context of the entire *E. coli* proteome, while residues below the *x*-axis are underrepresented. The red line corresponds to a *p*-value of 0.05. **(C)** The distribution of the BRI1 transphosphorylation sites in protein secondary structure. **(D)** The distribution of BRI1 transphosphorylation sites normalized for the total number of serine or threonine residues in a given secondary structure in the *E. coli* proteome.

**Figure 3:**
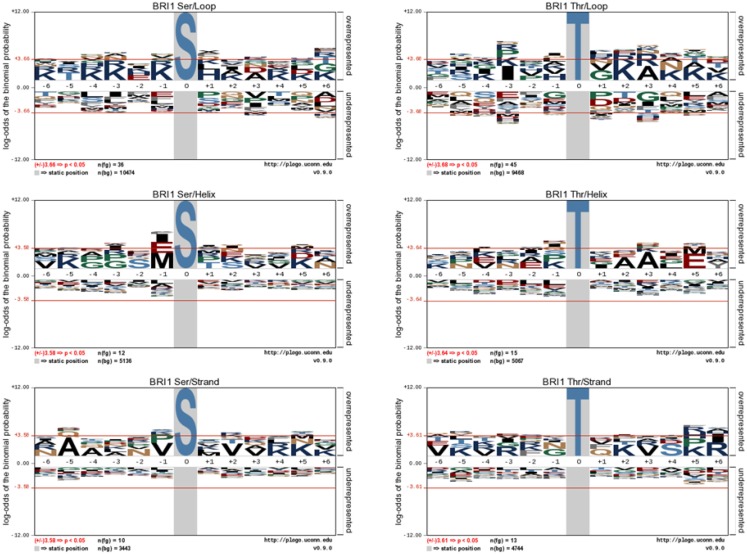
**pLogo motif analysis of BRI1 phospho substrates in *E. coli***. The substrates were categorized as their identity of Ser or Thr residues, and their localization in the protein secondary structure. The corresponding background database in *E. coli* proteome was used. The results revealed the distinct motif of loop substrates versus helix and strand substrates targeted by BRI1.

The phosphopeptides were analyzed in a Waters Q-Tof API-US Quad-ToF mass spectrometer interfaced with the Waters nanoAcquity UPLC system. Atlantis dC18 analytical column and Nanoease trap column, with a 60 min linear gradient of 1–60% acetonitrile in 0.1% formic acid were used. The peptides were analyzed in the data-dependent mode and the top four ions in each survey scan were selected for the tandem mass spectrometry analysis and subsequently excluded from MS/MS for 60 s. The raw data from mass spectrometry analysis was searched in Mascot limited to the taxonomy of *E. coli*, *Arabidopsis*, or soybean, and the auto-and transphosphorylation sites of the kinases were distinguished. The data were also searched in the decoy database in Mascot, and the false discovery rate of the peptides was less than 1%. The spectra for all phosphopeptides were manually examined. The complete list of the phosphopeptides identified following TiO_2_ or Fe^2+^-IMAC enrichment is presented in Table S1 in Supplementary Material, and autophosphorylation sites for the kinases are listed in Tables 1–4. Analysis of the phosphosites is presented in Figures 1–3 and 5–8.

**Table 1:** **Identified autophosphorylation sites of Flag-BRI1 expressed in *E. coli***.

Site	Counts	Mr (expt)	Mr (calc)	Expect	Peptides
T846	1	1027.1528	1027.4124	1.70E−03	R.TANN**pT**NWK.L
T846T851*	2	1605.2660	1605.6953	1.40E−04	R.TANN**pT**NWKL**pT**GVK.E
S858	283	1750.4736	1750.9018	1.70E−10	K.EAL**pS**INLAAFEKPLR.K
S906*	17	967.2228	967.4739	3.90E−06	K.DG**pS**AVAIKK.L
S917*	26	1160.2466	1160.5339	1.40E−02	K.LIHV**pS**GQGDR.E
T930*	6	1364.1704	1364.5393	1.30E−04	R.EFMAEME**pT**IGK.I
S963*	95	1451.3046	1451.6334	1.50E−05	K.YG**pS**LEDVLHDPK.K
S981T982*	6	935.0902	935.3303	3.60E−01	K.LNW**pSpT**R.R
S990*	2	894.2446	894.4688	1.90E−03	R.KIAIG**pS**AR.G
S1012*	7	1538.3170	1538.6977	1.50E−05	K.**pS**SNVLLDENLEAR.V
S1012 or S1013	255	1538.2560	1538.6977	1.40E−08	K.SSNVLLDENLEAR.V
S1012 and S1013*	55	1992.3800	1992.8264	4.50E−12	R.DMK**pSpS**NVLLDENLEAR.V
S1026*	26	961.1178	961.3729	6.50E−04	R.V**pS**DFGMAR.L
S1026	4	977.0876	977.3678	4.20E−03	R.V**pS**DFG(ox)MAR.L
S1109*	4	1641.3456	1641.7837	1.00E−05	K.LRI**pS**DVFDPELMK.E
S1109	2	1657.3358	1657.7786	4.60E−03	K.LRI**pS**DVFDPEL(ox)MK.E
T1147*	1	1517.2816	1517.6958	2.10E−04	R.RP**pT**MVQVMAMFK.E
T1147	6	1533.2764	1533.6907	2.60E−03	R.RP**pT**MVQVMA(ox)MFK.E
S1166	3	1640.2968	1640.7407	5.20E−08	K.EIQAGSGID**pS**QSTIR.S
T1169*	2	1640.2942	1640.7407	2.60E−08	K.EIQAGSGIDSQS**pT**IR.S
S1166T1169	61	1720.2422	1720.7070	7.40E−09	K.EIQAGSGID**pS**QS**pT**IR.S
S1168T1169	247	1720.2186	1720.7070	5.50E−09	K.EIQAGSGIDSQ**pSpT**IR.S

*Tryptic peptides identified to contain phosphorylated residues (pS or pT) are shown in bold and sites not reported unambiguously earlier are identified with asterisks. All spectra were subject to manual verification. The experimental (expt) and calculated (calc) monisotopic masses are shown along with the expectation value (Expect) reported in Mascot. The values for spectral counts for each identified peptide are the sums from three independent experiments. Oxidized methionine is identified as (ox)Met*.

### Motif analysis

Serine, threonine, and tyrosine phosphopeptides were mapped onto the *E. coli* proteome and extended (if necessary) to generate an aligned foreground data set of phosphorylated 13 mers, in which phosphorylation sites were always located at the central (0) position (phosphorylated residues within six residues of a protein terminus were discarded). Background data sets were created by taking all Ser/Thr/Tyr residues and their ±6 flanking residues in the *E. coli* proteome. In motif analyses of foreground peptides with a particular secondary structure, only peptides bearing the secondary structures specific to the analysis were used in the background. These aligned foreground and background data sets were used as inputs to analyze the phosphorylation motifs of each kinase using an internal prerelease version of the probability logo (pLogo) web software (v. 0.9.0, http://plogo.uconn.edu). Specifically, pLogos illustrate the log-odds binomial probability of each residue at each position in the foreground with respect to the background, where overrepresented residues are drawn above the *x*-axis and underrepresented residues are drawn below the *x*-axis. The most statistically significant residues are drawn closest to the *x*-axis, and a red horizontal bar is used to denote the 0.05 significance level (following Bonferroni correction). Examples of pLogos have been published previously (Chiang et al., 2008; Schwartz et al., 2009; Prisic et al., 2010).

## Results

### Auto- and transphosphorylation activities of BRI1 in *E. coli*

As noted above, previous studies reported that production of recombinant BRI1 resulted in increased phosphorylation of many *E. coli* proteins, whereas expression of the kinase-inactive directed mutant mBRI1 (K911E) did not (Oh et al., 2012). We have extended this observation in the present study, where *E. coli* extracts were digested with trypsin and phosphopeptides were enriched by IMAC (Fe^2+^) or TiO_2_ for a more thorough identification of BRI1 substrates. As a result, a total of 151 non-redundant phosphopeptides from 73 *E. coli* proteins were identified (Table S1 in Supplementary Material). Importantly, once again the *E. coli* extracts expressing the inactive mBRI1 (K911E) directed mutant were also analyzed with the same protocol but no phosphopeptides were found, suggesting that the 151 identified phosphopeptides were indeed substrates of the active BRI1 kinase. Because the expression level of many proteins in the *E. coli* proteome has been determined (Ishihama et al., 2008; Taniguchi et al., 2010), we could readily determine whether Flag-BRI1 was simply phosphorylating the most abundant bacterial proteins. Such analysis revealed that Flag-BRI1 phosphorylated substrates that vary by three orders of magnitude in protein amount in *E. coli* (Figure 1A). In addition to identifying phosphosites, we also quantified their relative abundance based on spectral count information (Zhang et al., 2009). The depth of the phosphopeptide recovery demonstrated the effectiveness of the phosphopeptide affinity enrichment protocols employed and the dynamic range of our mass spectrometry identification. Interestingly, there was no correlation between phosphopeptide abundance and the relative abundance of the corresponding protein in *E. coli* cells (Figure 1B). For example, the phosphopeptides with highest spectral counts in this study were phosphopeptide S453 from chaperone protein DnaK (K.**pS**LGQFNLDGINPAPR.G) and doubly phosphorylated peptide S21T28 from chaperone protein GroS (K.**pS**AGGIVL**pT**GSAAAK.S), but neither protein was in the top tier of protein abundance in *E. coli* (Ishihama et al., 2008; Taniguchi et al., 2010). Collectively, these results suggest that phosphorylation of specific sites on *E. coli* proteins likely reflected the inherent kinase properties of BRI1 and therefore could be used to further characterize the specificity of this important receptor kinase.

In addition to transphosphorylation, we also identified eighteen autophosphorylation sites of BRI1 kinase in *E. coli* (Table 1). Ten of these autophosphorylation sites had not been reported before (Ser-906, Ser-917, Thr-930, Ser-963, Ser-990, Ser-1012, Ser-1013, Ser-1026, Ser-1109, and Thr-1147), and all are located in the BRI1 kinase domain. Another three sites that were ambiguously identified earlier (Oh et al., 2000; Wang et al., 2005) now had spectral evidence to be supported as valid autophosphorylation sites for BRI1; the three residues were Thr-851 in the juxtamembrane domain, Ser-981 in the kinase domain, and Thr-1169 in the C-terminal domain of the kinase. In addition to site identification, we also estimated the relative abundance of each phosphopeptide, based on their spectral counts in mass spectrometry. Interestingly, three of the phosphopeptides (Ser-858, Ser-1012/Ser-1013, and Ser-1168/Thr-1169) accounted for 75% of the total BRI1 autophosphorylation phosphopeptides. The majority of these abundant phosphorylation sites were serine residues, which is consistent with the earlier observation that BRI1 autophosphorylated primarily on serine residues by total phosphoamino acid analysis of acid-hydrolyzed protein (Oh et al., 2000).

The 73 *E. coli* proteins phosphorylated by BRI1 are predicted to function in a range of diverse biological pathways (Figure 2A). Twenty-four are ribosomal proteins and many of them had more than one phosphorylation site identified. A number of proteins phosphorylated by BRI1 are involved in translation and protein folding. In addition, many transcription factors were also phosphorylated by BRI1, including lactose operon repressor (lacI), transcription termination factor Rho, and RNA polymerase subunits rpoA, rpoC, and rpoD, all of which are low abundance proteins. BRI1 also phosphorylated a number of enzymes in carbon metabolism, including *E. coli* glyceraldehyde-3-phosphate dehydrogenase A (gapA). To determine the sequence specificity for BRI1 trans- or autophosphorylation, the sequences surrounding sites of phosphorylation were analyzed. For transphosphorylation analysis, the phosphopeptides data sets, separated for phosphoserine and phosphothreonine sites, were analyzed against a corresponding peptide dataset for background probability calculations derived from the entire *E. coli* proteome (Schwartz et al., 2009). The resulting pLogo plots are shown in Figure 2B. In these plots, residue heights are proportional to their statistical significance in the context of the specified background, and residues above the *x*-axis are overrepresented while those below the *x*-axis are underrepresented. Though there was not a clear single motif that emerged from the transphosphorylation datasets, the analysis revealed that overrepresentation of basic residues (K,R) approached and/or exceeded statistical significance in a number of positions (−6, −3, −1, +5, +6 for Ser transphophorylation, and −4, −3, −1, +4, +5 for Thr transphosphorylation), indicating that BRI1 is a basophilic kinase. In contrast to the overrepresentation of basic residues, BRI1 may discriminate against aromatic residues and large hydrophobic residues (F, W, Y, L, M), especially at the positions −4, −1, and +3 for phosphoserine and −5, −3 for phosphothreonine, which was captured by motif analysis. In general, the motif for phosphoserine was similar to that for phosphothreonine, with the exception that phosphoserine sites had a stronger preference for basic residues at −6 and +6 positions, while phosphothreonine sites tended to favor lysine at −4 and alanine at the +3 position. The motif analysis indicated that the sequence surrounding the phosphorylatable residue was another determinant for the specificity of BRI1 transphosphorylation and was not simply promiscuous activity.

We also attempted to identify motifs associated with autophosphorylation of BRI1. For the autophosphorylation analysis, the phosphopeptide data sets, separated for phosphoserine and phosphothreonine sites, were analyzed individually. In contrast to the basophilic motifs observed for transphosphorylation activity with serine and threonine sites (Figure 2B, top panels), no distinct motifs were observed for autophosphorylation on either serine or threonine sites (Figure 2B, bottom panels). The exception was for autophosphorylation of threonine residues, where there was a clear preference for hydrophobic residues at +1 and +4, along with lysine at +3. Nonetheless, autophosphorylation and transphosphorylation motifs appear qualitatively different. One reason may stem from the fact that autophosphorylation sites are effectively present at a much higher concentration compared to transphosphorylation sites, and therefore restrictions based on specificity are less constraining. Alternatively, autophosphorylation can occur on sites that do not resemble substrate consensus sequences when the protein kinases are dimeric (even transiently) such that segments are exchanged between the two adjacent molecules (Oliver et al., 2007). An important point to note is that at least with BRI1, studies of autophosphorylation will not yield insights as to the transphosphorylation specificity of the kinase. However, it is important to note that in general, autophosphorylation sites of BRI1 identified *in vitro* tend to match those identified *in vivo*, validating the use of recombinant cytoplasmic domains for analysis of phosphorylation events (Shiu et al., 2004; Feuillet et al., 2011).

We further analyzed the BRI1-catalyzed phosphosites on *E. coli* proteins in terms of their localization in helices, loops, β-strands, and turns. As shown in Figure 2C, the majority of the BRI1-catalyzed transphosphorylation reactions on serine and threonine sites were localized in loop regions of *E. coli* proteins. However, such a distribution may be largely contributed by the higher number of serine and threonine residues in predicted loops in the *E. coli* proteome, because when normalized for the total number of serine and threonine residues in the different types of secondary structure, the frequency ratios (equivalent to percent of the total serine and threonine residues phosphorylated) were much more similar to one another (Figure 2D). To further characterize the kinase specificity for BRI1, the transphosphorylation phosphosites were divided based upon their localization in the secondary structure, and the motifs associated with loops, helices, and β-strands were analyzed individually for phosphoserine and phosphothreonine sites (Figure 3). Interestingly, the motifs associated with phosphosites in the secondary structures were different from one another. While the loop motif recapitulated the preference for basic residues, those features were less apparent for helices and strands. In contrast, phosphoserine sites in helices showed some preference for hydrophobic residues at several positions (in particular −1), while phosphothreonine residues in helices had a preference for alanine at +3 and glutamate at +5. Moreover, the phosphorylation motifs for phosphosites in strands appeared to be somewhat intermediate between the loop and helix motifs (Figure 3). Our analysis used as the background database the serine and threonine sequences of the corresponding secondary structures, which took into account the distribution of different amino acid residues in the secondary structure. Therefore, the differences in the motifs for loops and helices were likely caused by the inherent kinase specificity of BRI1. Thus, secondary structure of the substrate protein emerges as another factor to consider when assessing potential target proteins. To summarize, the *E. coli* transphosphorylation assay identified many bacterial proteins as substrates for BRI1 and enabled the characterization of BRI1 kinase specificity in greater detail.

### Phosphorylation specificity of BAK1, PEPR1, FLS2, and CDPKβ

**Figure 4:**
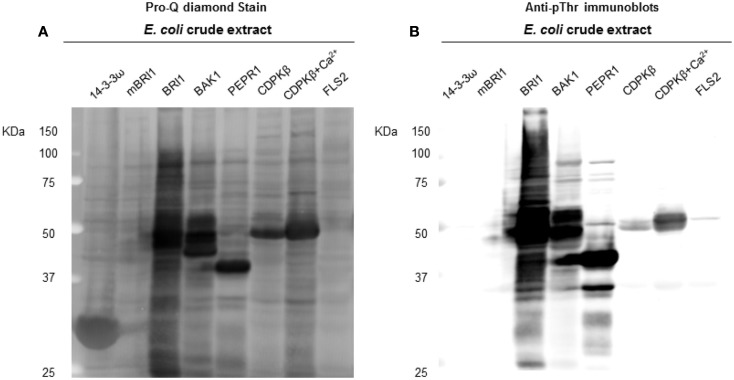
**Transphosphorylation of *E. coli* proteins by the protein kinases tested in the present study**. **(A)** ProQ diamond stained blot showing the increase of overall phosphorylation of *E. coli* proteins, when exogenous kinases were expressed. LRK non-RD-type kinase FLS2, BRI1 kinase dead mBRI1 (K911E), and the non-kinase protein 14−3−3ω were used as controls. In addition to RLK RD kinases (BRI1, BAK1, and PEPR1), kinase CDPKβ was also found with considerable increase in *E. coli* phosphorylation, when a 1 mM Ca^2+^ was added to *E. coli* growth culture. **(B)** Anti-pThr immunoblots confirmed the phospho bands identified by ProQ.

**Figure 5:**
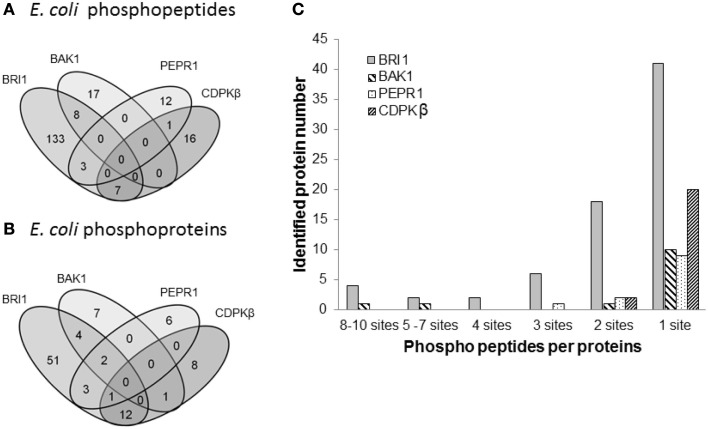
**Comparison of transphosphorylation of *E. coli* proteins for BRI1, BAK1, PEPR1, and CDPKβ**. **(A)** Venn diagram illustrating the overlap in phosphopeptides and **(B)** phosphoproteins identified as substrates of the four kinases expressed individually in *E. coli*. **(C)** Number of phosphosites per protein that were identified following expression of the protein kinases.

**Figure 6:**
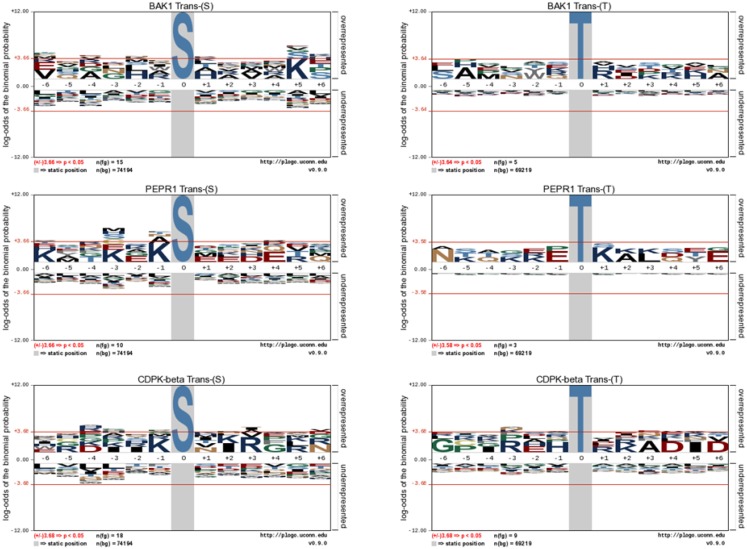
**Motif analysis for BAK1, PEPR1, and CDPKβ transphosphorylation of *E. coli* proteins**. pLogo motif analysis of transphosphorylation on serine (left panels) and threonine (right panels) sites in *E. coli*. The number of sites analyzed is indicated in each panel. The phosphorylated residue is annotated as position 0, and the six upstream or downstream residues are annotated as −6 to −1 and +1 to +6, respectively. Residues above the *x*-axis are overrepresented, relative to their statistical significance in the context of the entire *E. coli* proteome, while residues below the *x*-axis are underrepresented. The red line corresponds to a *p*-value of 0.05.

Following the characterization of BRI1 kinase specificity, three other receptor kinases (BAK1, PEPR1, and FLS2) and one soluble protein kinase (CDPKβ) were analyzed for transphosphorylation of *E. coli* proteins. All are RD-type protein kinases with the exception of FLS2, which is a non-RD-type kinase. As with BRI1, the receptor kinases that were tested consisted of their cytoplasmic domains with an N-terminal Flag-tag fusion. Full-length CDPKβ was expressed with an N-terminal 6xHis-tag. We confirmed that all four kinases were expressed at generally similar levels to BRI1 in *E. coli*. For example, average yields of recombinant protein kinases from 100 mL of cultured *E. coli* cells were: 140 μg BRI1, 150 μg BAK1, 130 μg PEPR1, 100 μg FLS2, and 170 μg CDPKβ. Figure 4 compares the phosphorylation of *E. coli* proteins during expression of the five protein kinases tested in the present study with *E. coli* cells expressing the non-kinase proteins, 14−3−3ω and EF1A, or the kinase-inactive mBRI1, serving as negative controls. It is important to note that ProQ Diamond does stain non-phosphorylated proteins as well, which is apparent with the gray staining of the large amount of 14−3−3ω protein in Figure 4A. However, phosphorylation of numerous bacterial proteins is readily apparent when active kinases are expressed or when phosphothreonine-containing proteins are detected by immunoblotting (Figure 4B). Because the CDPKs are calcium-dependent kinase, in preliminary experiments we compared *E. coli* cells expressing CDPKβ in standard media or media containing 1 mM CaCl_2_. The addition of exogenous Ca^2+^ clearly enhanced transphosphorylation activity of CDPKβ activity *in situ* as evidenced by increased staining of numerous *E. coli* proteins with ProQ Diamond phosphoprotein stain. Therefore, for all subsequent studies of CDPKβ-mediated transphosphorylation, *E. coli* cells grown in the presence of 1 mM CaCl_2_ were used as the source of bacterial proteins for phosphosite analysis. Exogenous calcium in the growth medium also increased autophosphorylation of CDPKβ (ProQ-stained protein band at ∼50 kDa). With the non-RD-type kinase, FLS2, there was little evidence for either transphosphorylation or autophosphorylation of the protein (Figure 4). Consistently, we did not identify any autophosphorylation sites when the purified Flag-FLS2 protein was analyzed by LC-MS/MS and only one putative transphosphorylation site was found, corresponding to Ser-113 in isocitrate dehydrogenase (icd) [NADP] (Table S1 in Supplementary Material). However, Ser-113 of icd is an endogenous phosphorylation site in *E. coli* (Hurley et al., 1990), and hence cannot be attributed to FLS2. In contrast, 25 transphosphorylation sites on 13 *E. coli* proteins were identified during expression of BAK1, 16 transphosphorylation sites on 12 *E. coli* proteins were identified during expression of PEPR1, and 24 phosphopeptides on 22 proteins were transphosphorylated by CDPKβ in *E. coli* (Table S1 in Supplementary Material). While a few phosphosites (Figure 5A) and proteins (Figure 5B) were common among BRI1, BAK1, PEPR1, and CDPKβ, the majority of phosphosites were specific for the individual kinases, and this is reflected in the similar but distinct phosphorylation motifs that were identified for the kinases (Figure 6). A prominent feature for all of the motifs was a preference for basic residues at one or more positions. For example, BAK1 phosphorylation tended to prefer a lysine residue at +5, PEPR1 preferred basic residues at −6, −3, and −1, and CDPKβ preferred basic residues at −3, −1, and +3. Clearly all are basophilic kinases, with potentially some differences once again between sites of serine and threonine phosphorylation. Recombinant CDPKβ has been studied in the past in terms of synthetic peptide specificity, and several distinct motifs were identified that involve altered positioning of basic and hydrophobic residues (Huang and Huber, 2001; Huang et al., 2001; Sebastià et al., 2004); the motif presented in Figure 6 for CDPKβ appears to be something of a composite of those different motifs.

**Figure 7:**
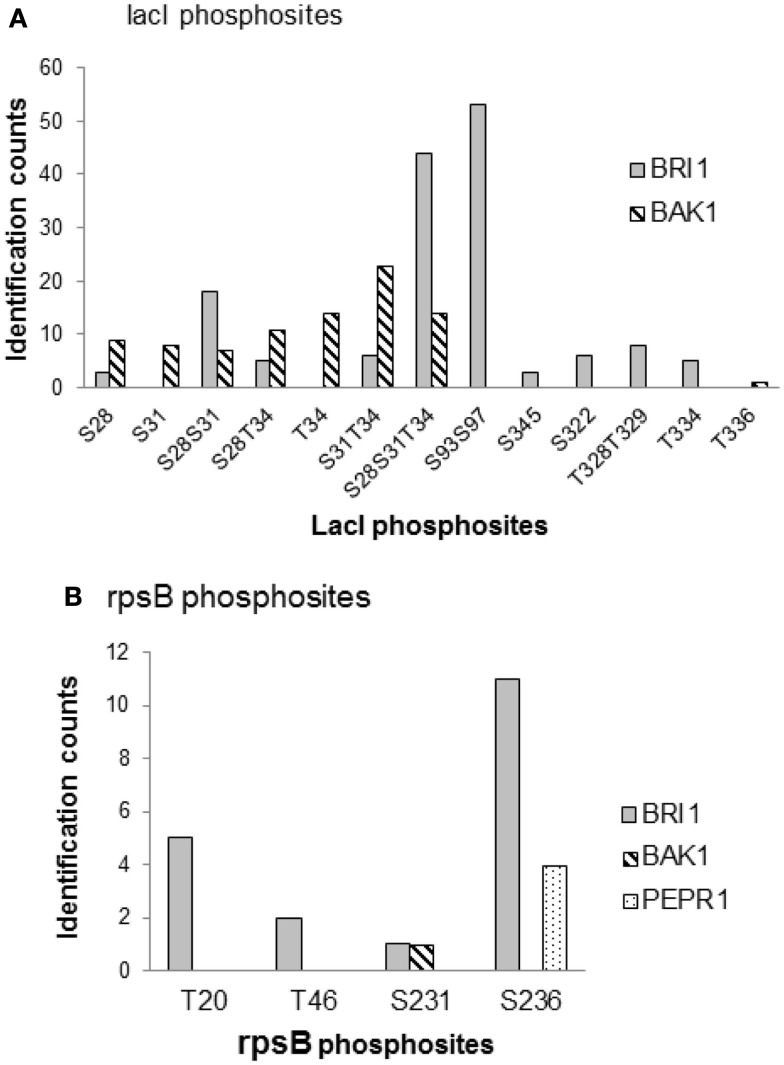
**Comparison of kinase specificities for BRI1, BAK1, and PEPR1 with lactose operon repressor (lacI) or 30S ribosomal protein S2 (rpsB) as substrate**. **(A)** Abundance of specific phosphopeptides of lacI and **(B)** ribosomal protein rpsB that were transphosphorylated by the indicated protein kinases expressed in *E. coli* cells. The relative abundance of each phosphopeptide species was based on the spectral counts in the mass spectrometry identification, and reflects the sum of four independent experiments involving two TiO_2_ and two IMAC (Fe^2+^) enrichment steps. Note that there was no evidence for phosphorylation of lacI by PEPR1, or rpsB by PEPR1 or CDPKβ, and that phosphopeptides are identified using the single letter abbreviations for Ser (S) and Thr (T).

To further compare the kinase specificities of BRI1 and BAK1, the phosphorylation patterns for two common substrates – the lactose operon repressor (lacI) and 30S ribosomal proteins S2 (rpsB) – were analyzed in more detail (Figure 7). LacI is a transcription factor in *E. coli*, and was phosphorylated by BRI1 on 10 peptide species and by BAK on 8 peptide species in the transphosphorylation assay (Table S1 in Supplementary Material). The relative abundance of the phosphorylated peptides was estimated by spectral counting for further comparison (Figure 7A). Interestingly, the distribution of the relative abundance of phosphopeptide species was dramatically different for BRI1 and BAK1. For BRI1 transphosphorylation, three multiply phosphorylated peptide species (S93S97, S28S31T34, and S28S31) were phosphorylated to a greater extent compared to the other phosphopeptides. In contrast, the phosphorylation abundance for the peptide species was more evenly distributed for BAK1 transphosphorylation, with the doubly phosphorylated species S31T34 having the highest spectral counts. While the S31T343 phosphopeptide was also phosphorylated by BRI1, it was a relatively minor peptide species consistent with the notion that spectral counting reflects specificity of the kinase being expressed rather than simply factors that affect detection of the species in the MS analysis. Moreover, while several peptides species were commonly phosphorylated by both BRI1 and BAK1, several peptide species (S93S97, S345, S322, T328T329, and T334) were specifically phosphorylated by BRI1, whereas others (S31, T34, and T336) were specifically phosphorylated by BAK1 (Figure 7A). Thus, the phosphorylation patterns on lacI confirmed the overlapping but distinct kinase specificities of BRI1 and BAK1. Similarly, the phosphorylation patterns of another common substrate, rpsB, were markedly different among kinases BRI1, BAK1, and PEPR1 (Figure 7B). BRI1 phosphorylated rpsB on four different peptide species corresponding to phosphosites T20, T46, S231, and S236, with S236 and T20 recording the highest spectral counts. In contrast, BAK1 only phosphorylated rpsB at the Ser-231 site, while PEPR1 only phosphorylated rpsB on the Ser-236 site. Such distinct phosphorylation patterns on rpsB again reflected the overlapping but distinct kinase specificities for the three receptor kinases BRI1, BAK1, and PEPR1, but may also simply reflect the lower level of transphosphorylation catalyzed by BAK1 and PEPR1 compared to BRI1.

In addition to catalyzing the transphosphorylation of *E. coli* proteins, both BAK1 and PEPR1 were also autophosphorylated on a number of residues. For BAK1, 11 autophosphorylation sites were identified in *E. coli*, including six autophosphorylation sites that had not been reported previously (Table 2). These new BAK1 autophosphorylation sites were Ser-324, Tyr-443, Ser-465, and Thr-466, and all are located in the BAK1 kinase domain. From the spectral counting analysis, phosphopeptides T455, T446T449, and T446T449T450 were highest in abundance. Interestingly, these residues are all in the activation segment of the enzyme and are all phosphothreonine residues. This result suggested the higher relative abundance of BAK1 autophosphorylation on threonine residues, which is in contrast to BRI1 autophosphorylation that occurred primarily on serine residues (Table 1; Oh et al., 2012). For PEPR1, seven autophosphorylation sites were identified for the first time (Table 3). These sites included three tyrosine autophosphorylation sites (Tyr-805, Tyr-842, and Tyr-910) and several sites of serine and threonine phosphorylation. Thus, PEPR1 can also be classified as a dual specificity kinase similar to BRI1 and BAK1 (Oh et al., 2009, 2010). The Tyr-842 site is located within the conserved ATP binding region 833–843 (GRGAHGIVYR), and phosphorylation on this site might be expected to directly affect ATP binding, and will be interesting to follow up in future studies. Other autophosphorylation sites of PEPR1 included Thr-808 in the juxtamembrane domain, and Ser-848, Ser-861, and Ser-868 in the kinase domain. Curiously, the spectral counting recorded that the phosphopeptide containing Tyr-805 (Y805 in Table 3) was by far the highest in abundance, suggesting a relative higher stoichiometry on tyrosine autophosphorylation for PEPR1 compared to serine/threonine autophosphorylation. It is also worth noting that Tyr-805 is likely to be the first autophosphorylation site within the juxtamembrane domain, and thus is similarly positioned to Tyr-831 in BRI1, and will be interesting to study by directed mutagenesis in the future. CDPKβ also autophosphorylated on a number of residues but a complete description requires additional experimentation and will be reported elsewhere.

**Table 2:** **Identified autophosphorylation sites of Flag-BAK1 expressed in *E.coli***.

Site	Counts	Mr (expt)	Mr (calc)	Expect	Peptide
S286	2	1430.4072	1430.6079	8.40E−05	R.ELQVA**pS**DNFSNK.N
S290	10	1430.4140	1430.6079	2.00E−06	R.ELQVASDNF**pS**NK.N
S286 and S290	5	2063.6382	2063.9078	2.80E−10	R.ELQVA**pS**DNF**pS**NKNILGR.G
T324*	6	2469.7507	2470.1022	4.20E−13	R.**pT**QGGELQFQTEVEMISMAVHR.N
T324	5	2485.7923	2486.0971	2.00E−12	R.**pT**QGGELQFQTEVE(ox)MISMAVHR.N
T324	2	2501.7946	2502.0920	2.60E−07	R.**pT**QGGELQFQTEVE(ox)MIS(ox)MAVHR.N
T446	9	1078.1902	1078.4808	5.50E−03	K.D**pT**HVTTAVR.G
T446	1	1728.6043	1728.7906	2.50E−04	K.LMDYKD**pT**HVTTAVR.G
T449	31	1078.2848	1078.4808	4.60E−05	K.DTHV**pT**TAVR.G
T450	2	1078.3472	1078.4808	2.00E−03	K.DTHVT**pT**AVR.G
T446T449	4	1158.3110	1158.4472	3.20E−03	K.D**pT**HV**pT**TAVR.G
T446T449	207	1808.5267	1808.7569	2.30E−09	K.LMDYKD**pT**HV**pT**TAVR.G
T446T449	43	1824.4798	1824.7519	9.30E−09	K.L(ox)MDYKD**pT**HV**pT**TAVR.G
T446T450	9	1158.2466	1158.4472	3.10E−04	K.D**pT**HVT**pT**AVR.G
T446T450	6	1808.3242	1808.7569	1.20E−05	K.LMDYKD**pT**HVT**pT**AVR.G
T446T450	1	1824.3166	1824.7519	3.70E−03	K.L(ox)MDYKD**pT**HVT**pT**AVR.G
T449T450	11	1158.2642	1158.4472	1.30E−04	K.DTHV**pTpT**AVR.G
T446T449T450	1	1238.2236	1238.4135	3.10E−02	K.D**pT**HV**pTpT**AVR.G
T446T449T450	111	1888.5010	1888.7233	3.70E−08	K.LMDYKD**pT**HV**pTpT**AVR.G
T446T449T450	1	1904.4894	1904.7182	1.10E−05	K.LMDYKD**pT**HV**pTpT**AVR.G
Y443T449*	5	1808.5414	1808.7569	3.00E−07	K.LMD**pY**KDTHV**pT**TAVR.G
Y443T449	3	1824.2875	1824.7519	3.60E−03	K.L(ox)MD**pY**KDTHV**pT**TAVR.G
Y443T449T450	17	1888.4788	1888.7233	1.10E−04	K.LMD**pY**KDTHV**pTpT**AVR.G
Y443T446T450	1	1888.5172	1888.7233	2.90E−03	K.LMD**pY**KD**pT**HVT**pT**AVR.G
T455	400	1622.3617	1622.7705	2.80E−10	R.G**pT**IGHIAPEYLSTGK.S
T455S465*	48	1702.5668	1702.7368	1.10E−07	R.G**pT**IGHIAPEYL**pS**TGK.S
T455T466*	37	1702.5156	1702.7368	1.20E−06	R.G**pT**IGHIAPEYLS**pT**GK.S

*Tryptic peptides identified to contain phosphorylated residues (pS, pT, or pY) are shown in bold and sites not reported unambiguously earlier are identified with asterisks. All spectra were subject to manual verification. The experimental (expt) and calculated (calc) monisotopic masses are shown along with the expectation value reported in Mascot. The values for spectral counts for each identified peptide are the sums from three independent experiments. Oxidized methionine is identified as (ox)Met*.

**Table 3:** **Identified autophosphorylation sites of Flag-PEPR1 expressed in *E.coli***.

Site	Counts	Mr (expt)	Mr (calc)	Expect	Peptide
Y805*	110	2002.4556	2002.9289	8.90E−10	K.DA**pY**VFTQEEGPSLLLNK.V
T808*	15	2002.4616	2002.9289	9.50E−10	K.DAYVF**pT**QEEGPSLLLNK.V
Y842*	3	951.2092	951.4328	6.10E−04	R.GAHGIV**pY**R.A
S848*	9	1258.3030	1258.6322	7.10E−08	R.ASLG**pS**GKVYAVK.R
S861*	5	1021.2626	1021.5110	9.00E−05	R.LVFA**pS**HIR.A
S868*	1	916.1130	916.3296	1.40E−01	R.ANQ**pS**MMR.E
Y910*	8	1450.3386	1450.6857	2.00E−06	K.GSL**pY**DVLHGVSPK.E

*Tryptic peptides identified to contain phosphorylated residues (pS, pT, or pY) are shown in bold and sites not reported unambiguously earlier are identified with asterisks. All spectra were subject to manual verification. The experimental (expt) and calculated (calc) monisotopic masses are shown along with the expectation value reported in Mascot. The values for spectral counts for each identified peptide are the sums from three independent experiments. Oxidized methionine is identified as (ox)Met*.

**Figure 8:**
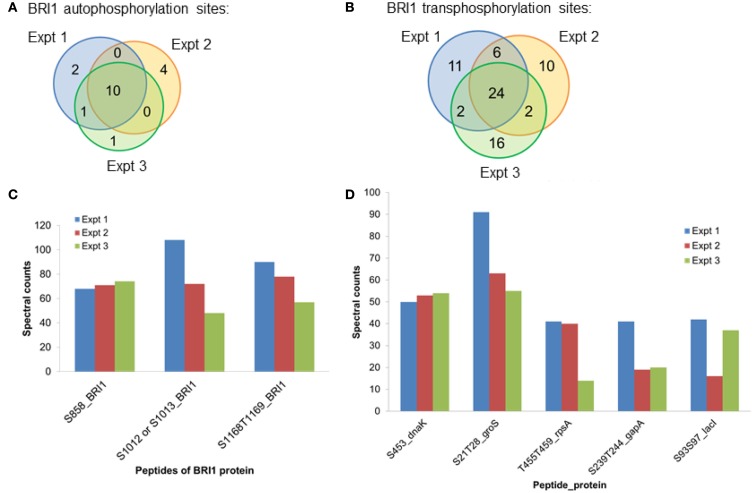
**The reproducibility of the proteomics analysis in this study**. Three biological replicates of BRI1 auto- and transphosphorylated *E. coli* cells were digested with trypsin. Phosphopeptides were enriched with the TiO_2_ method, before identification with data-dependent analysis in the mass spectrometry. **(A)** Venn diagram showing the overlap of the identified BRI1 autophosphorylation peptides from three biological replicates. Ten abundant BRI1 autophosphorylation peptides were commonly identified. **(B)** Venn diagram showing the overlap of the identified BRI1 transphosphorylation peptides from three biological replicates. Twenty-four abundant BRI1 transphosphorylation peptides were commonly identified. **(C)** The reproducibility of spectral counts of three most abundant BRI1 autophosphorylation peptides using TiO_2_ enrichment. **(D)** The reproducibility of spectral counts on the five most abundant BRI1 transphosphorylation peptides using TiO_2_ enrichment. Note that phosphopeptides are identified using the single letter abbreviations for Ser (S) and Thr (T).

Spectral counting was used to quantify the relative abundance of phosphorylation of phosphosites (Mueller et al., 2008). This method was effective in a linear dynamic range over two orders of magnitude (Liu et al., 2004), but due to the stochastic nature of the sampling process for data-dependent mass spectrometry, the spectral counting quantification for the abundant peptides was more reliable than for the low abundance peptides (Mueller et al., 2008; Zhang et al., 2009). Therefore, we focused on the higher abundance phosphopeptides for the spectral quantification. In our experiments, we also found good reproducibility among independent experiments in sampling the abundant phosphopeptides. For example, in the case of BRI1, we identified a core of 10 abundant autophosphorylation peptides and 24 abundant transphosphorylation peptides from three biological replicates. The top three autophosphorylation peptides and top five transphosphorylation peptides had good consistency in their spectral quantification (Figure 8). The separation of the higher abundance autophosphorylation peptides from the lower abundance autophosphorylation peptides was more than an order of magnitude in our analyses. Thus, phosphopeptides containing phosphorylation sites Ser-858, Ser-1012, Ser-1013, Ser-1168, and Thr-1169 for BRI1 (Table 1); Thr-446, Thr-449, and Thr-455 for BAK1 (Table 2); and Tyr-805 for PEPR1 (Table 3), were identified as most abundant phosphopeptides, and their corresponding sites were considered as major phosphorylation sites for the proteins.

## Discussion

In this study, we demonstrate that monitoring the transphosphorylation of *E. coli* proteins during production of recombinant protein kinases provides a simple and robust system to characterize kinase activity and more importantly, specificity. We predict that the *E. coli* transphosphorylation assay system will have broad application to the study of monomeric kinases that activate by autophosphorylation or do not require autophosphorylation for activity. While many studies have expressed recombinant protein kinases in *E. coli* and in some cases demonstrated that autophosphorylation occurred within the bacterial cells, to our knowledge there are only a few reports that bacterial proteins are concurrently phosphorylated. Recently, we reported for the first time that expression of the cytoplasmic domain of BRI1 in *E. coli* resulted in the transphosphorylation of numerous bacterial proteins, and 77 phosphosites were identified by LC-MS/MS analysis (Oh et al., 2012). In that study we demonstrated that co-expression of calmodulin with BRI1 attenuated the autophosphorylation of BRI1 and hence reduced activation of the kinase, which provided new insights into crosstalk between calcium and BR signaling. However, it was not clear whether the specific sites phosphorylated on the bacterial proteins provided meaningful insights into the intrinsic kinase specificity of BRI1 and in the present study, we demonstrate that is indeed the case. We used the recombinant cytoplasmic domain of BRI1 (and other receptor kinases), because previous comparisons between BRI1 (and also BAK1) in terms of *in vitro* versus *in vivo* autophosphorylation sites showed a general overlap among the sites (Wang et al., 2005, 2008). Moreover, while extracellular domains would be expected to affect kinase activity it is less likely that they would influence kinase specificity. Thus, we believe that studies with the cytoplasmic domains of receptor kinases can provide useful insights regarding the intrinsic specificity of the full-length proteins.

Transphosphorylation of *E. coli* proteins by BRI1 was determined to be specific based on several lines of evidence. First, the bacterial proteins phosphorylated were not simply the most abundant ones (Figure 1A). Second, the abundance of specific phosphopeptides was not related to the abundance of the parent protein (Figure 1B). Third, a motif was identified where the phosphorylated residue was bracketed by basic residues at several positions both N- and C-terminal to the serine/threonine (Figure 2B). Fourth, expression of four other protein kinases resulted in the phosphorylation of a distinct set of *E. coli* proteins and specific residues (Figure 5), establishing kinase-distinct motifs (Figure 6). Collectively, these lines of evidence suggest that the results obtained provide meaningful insights to the intrinsic specificity of the recombinant protein being expressed in *E. coli* and has the potential to be of broad applicability to the study of various protein kinases from diverse organisms that are presently not well characterized.

With respect to the phosphorylation motif identified for BRI1 (Figure 2B), there are several points that are worth mentioning. First, the basophilic motifs identified for transphosphorylation of *E. coli* proteins on serine and threonine residues (Figure 2B, top panels) are reminiscent of the earlier results obtained with synthetic peptide substrates. BRI1 will phosphorylate the SP11 peptide (sequence: GRJRRIASVEJJKK, where J is norleucine and the underlined serine is the phosphorylated residue; Oh et al., 2000), which is derived from the regulatory phosphorylation site in spinach sucrose phosphate synthase (McMichael et al., 1993). Studies with peptide variants of SP11 established that the hydrophobic residue at +4 and the basic residues at the +6, −4, −3, +5, and +6 positions function as positive recognition elements because individual substitution with alanine dramatically decreased peptide kinase activity. These results are generally consistent with the motifs derived from analysis of transphosphorylation of *E. coli* proteins that BRI1 preferentially phosphorylates serine and threonine residues flanked with basic residues at both downstream and upstream positions (Figure 2B). However, it is interesting that a role for a hydrophobic residue at the +4 position (only position tested) with synthetic peptide substrates, was not observed for phosphosites in protein substrates. A role for basic residues is also apparent in the putative in planta substrates of BRI1, including BKI1 (Jaillais et al., 2011; Wang et al., 2011), BSK1 (Tang et al., 2008), TRIP-1 (Ehsan et al., 2005), and BAK1 (Wang et al., 2008), which are phosphorylated at sites that are similarly surrounded by basic residues (Table 4). Thus it is still clear that BRI1 is a basophilic kinase based on the phosphosites targeted in plant, bacterial, and peptide substrates. The present findings provide the first broad assessment of the intrinsic ability of BRI1 to transphosphorylate a wide variety of proteins and reveal new insights relative to those obtained with previous (and more limited scale) synthetic peptide studies.

**Table 4:** **Potential BRI1-mediated transphosphorylation sites in *Arabidopsis* substrate proteins**.

Substrate	Site	Position	Reference
		6 3 0 3 6	
BKI1	Y211	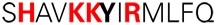	Jaillais et al. (2011)
	S270	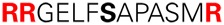	Wang et al. (2011)
	S274	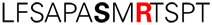	Wang et al. (2011)
BSK1	S230	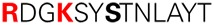	Tang et al. (2008)
TRIP-1	T14	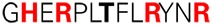	Ehsan et al. (2005)
	T89	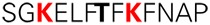	
	T197/198	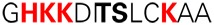	
BAK1	S290	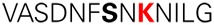	Wang et al. (2008)
	T312	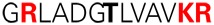	
	T446	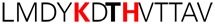	
	T449	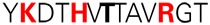	
	T450	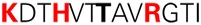	
	T455	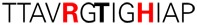	

**Arabidopsis* proteins that have been shown or suggested to be BRI1 substrates with phosphosites identified by mutagenesis or LC-MS/MS analysis*.

The transphosphorylation motifs identified for the four active kinases studied in the present report are summarized in Table 5, which shows positions at which basic residues (K,R) approached and/or exceeded statistical significance relative to background probability. Interestingly, there was no evidence for overrepresentation of hydrophobic residues at specific positions surrounding the phosphorylated residue. That observation is particularly significant with respect to the phosphoserine motif targeted by CDPKβ, because in contrast to the other kinases, there is some background information on synthetic peptide substrates of this kinase. Studies with CDPKβ (Hardin et al., 2009) have documented phosphorylation of peptides corresponding to the classic motif: Φ-x-[KR]-x-x-[ST]-x-x-x-Φ, where Φ is a hydrophobic residue, and also the non-classical motifs [KR]-Φ-x(4)-[ST]-x-[KR], referred to as the ACA2 motif (Huang et al., 2001), and [KR]-Φ-[ST]-Φ-x-[KR]-[KR], referred to as the ACS motif (Sebastià et al., 2004). These motifs are distinguished on the basis of the positioning of both basic and hydrophobic residues. It is noteworthy that with protein substrates, a clear role for hydrophobic residues was not observed whereas basic residues were prominent both N- and C-terminal to the phosphorylated serine. Thus, results with the bacterial transphosphorylation system have provided new insights to the intrinsic activity of CDPKβ with diverse protein substrates. In contrast, nothing was known about the kinase activity of PEPR1, but as demonstrated in the present study, PEPR1 preferentially targets serine residues flanked by basic residues at −1, −3, and −6. This is similar to the BRI1 motif, with the exception that there is no preference for basic residues at +5 and +6. BAK1 displayed the simplest motif for phosphoserine sites, with overrepresentation of basic residues only at the +5 position.

**Table 5:** **Summary of preference for basic residues (B) surrounding serine and threonine phosphosites in bacterial proteins targeted by recombinant protein kinases expressed in *E. coli***.

Protein kinase	Position													Position												
	−6	−5	−4	−3	−2	−1	0	1	2	3	4	5	6	−6	−5	−4	−3	−2	−1	0	1	2	3	4	5	6
BRI1	**B**	X	X	**B**	X	**B**	**S**	X	X	X	X	**B**	**B**	X	X	**B**	**B**	X	**B**	**T**	X	X	X	**B**	**B**	X
BAK1	X	X	X	X	X	X	**S**	X	X	X	X	**B**	X	X	X	X	X	X	X	**T**	X	X	X	X	X	X
PEPR1	**B**	X	X	**B**	X	**B**	**S**	X	X	X	X	X	X	X	X	X	X	X	X	**T**	**B**	X	X	X	X	X
CDPKβ	X	X	X	X	X	**B**	**S**	X	X	**B**	X	X	X	X	X	X	**B**	X	X	**T**	X	**B**	X	X	X	X

*Positions where basic residues (K,R) approached and/or exceeded statistical significance in the pLogo plots for BRI1 (Figure 2B) and BAK1, PEPR1, and CDPKβ (Figure 6) are shown in the summary plot*.

We also characterized the autophosphorylation of BRI1, BAK1, and PEPR1 in *E. coli* and identified a number of new autophosphorylation sites were for each receptor kinase (Tables 1– 3). Some of the autophosphorylation sites were identified earlier and their function has been studied by mutagenesis. For example, substitution with alanine to produce the S858A and S1168A directed mutants of BRI1 had no effect on overall autophosphorylation but reduced peptide kinase activity *in vitro* (Wang et al., 2005), and the T455A mutant of BAK1 significantly reduced the overall autophosphorylation of the kinase *in vitro* (Wang et al., 2008), consistent with the notion that these phosphosites may be essential for kinase activity. It will be interesting to test the function of some of the newly identified autophosphorylation sites via directed mutagenesis in future studies. It is also interesting that several of the known autophosphorylation sites of BRI1 and BAK1 (Wang et al., 2005, 2008; Karlova et al., 2009) were not identified in our analyses. The basis for this is not clear, but it is possible that some sites are phosphorylated *in vitro* but not *in situ* in *E. coli* cells (as utilized in the present study) or that our protocol was biased toward the identification of major phosphorylation sites and we were not detecting the lower abundance sites. This is an interesting question to explore in the future, but regardless our results add substantially to the characterization of *in vitro* autophosphorylation sites for BRI1, BAK1, and PEPR1 and provide new sites for future functional studies.

Another interesting aspect about the phosphorylation of BRI1 was that the autophosphorylation motif of BRI1 was very different from the transphosphorylation motif (Figure 2B). The event of phosphorylation usually requires two critical elements: the recruitment of the substrate and the site specificity of the kinase (Zhu et al., 2005). However, when the concentrations of the substrates are very high (as the case of autophosphorylation), the selectivity of amino acid sequence for phosphorylation by the kinase can be diluted (Zhu et al., 2005). For example, protein kinase C alpha (PKC-α) is a kinase that strongly preferred basic residues in phosphorylation, but PKC-α autophosphorylates on Thr-638, which is not flanked by basic residues (Keranen et al., 1995; Bornancin and Parker, 1996). An alternative explanation is that activation segment exchange may occur when a kinase dimerizes (even transiently) allowing for autophosphorylation on non-consensus substrate sites (i.e., sites not flanked by basic residues; Pike et al., 2008). That this might be occurring with BRI1 is further suggested by the observation that the full cytoplasmic domain of BRI1 exists as a dimer in solution, but when the juxtamembrane domain is removed the protein is monomeric (Jaillais et al., 2011) and autophosphorylation of kinase domain and carboxy terminal domain residues is dramatically reduced (Oh et al., 2009). Taken together the results suggest that the juxtamembrane domain is essential for BRI1 dimerization, which we speculate allows for activation segment exchange and autophosphorylation on non-consensus substrate sites. As a result, the kinase domain activates and presumably then allows for autophosphorylation at additional non-consensus sites outside of the activation segment. This provides a plausible explanation for the observation that the autophosphorylation motif for BRI1 did not mirror its transphosphorylation motif.

To conclude, we have characterized a convenient and effective system – the *E*. coli transphosphorylation assay – to study the kinase activity and specificity of recombinant protein kinases. Advantages of the system include the fact that it is an *in situ* rather than *in vitro* assay (as in the case of peptide kinase assays), and therefore is a step closer to the *in vivo* situation where phosphorylation reactions occur in the context of protein complexes and cellular complexities. Since endogenous protein phosphorylation is dramatically reduced in bacterial cells, the problem of kinase redundancy is eliminated and it is much simpler to link specific substrates with the kinase being expressed. Disadvantages include the fact that it is a heterologous system and the bacterial protein substrates may be very different from the real plant substrates. However, it can provide insights into the intrinsic specificity of protein kinases, which may be of particular importance for kinases that are not well characterized. Indeed, we are aware of another manuscript in review that demonstrates the phosphorylation of *E. coli* proteins during expression of human basophilic (Protein Kinase A) and acidophilic (Casein Kinase II) kinases (D. Schwartz, personal communication). Importantly, the bacterial proteins phosphorylated reflected the well-established motifs targeted by both animal kinases. These results, coupled with the current study that focused on plant kinases, provide strong proof of concept that the *E. coli* transphosphorylation assay can be applied broadly to protein kinases of different families and organisms. Indeed, we are aware of another manuscript in review that demonstrates the phosphorylation of *E. coli* proteins during expression of human basophilic (Protein Kinase A) and acidophilic (Casein Kinase II) kinases (D. Schwartz, personal communication). Importantly, the bacterial proteins phosphorylated reflected the well-established motifs targeted by both animal kinases. These results, coupled with the current study that focused on plant kinases, provide strong proof of concept that the *E. coli* transphosphorylation assay can be applied broadly to protein kinases of different families and organisms.

## Conflict of Interest Statement

The authors declare that the research was conducted in the absence of any commercial or financial relationships that could be construed as a potential conflict of interest.

## Supplementary Material

The Supplementary Material for this article can be found online at (http://www.frontiersin.org/Plant_Physiology/10.3389/fpls.2012.00262/abstract).

Supplementary Table S1 **Identification of transphosphorylation sites of *E. coli* proteins mediated by BRI1, BAK1, PEPR1, CDPKβ, or FLS2**. Tryptic peptides containing phosphorylated residues (pS or pT) are listed along with the experimental (expt) and calculated (calc) monoisotopic masses and the expectation value (expect) reported in Mascot. The values for spectral counts for each phosphopeptide are the sums from three independent experiments. Values for the abundance of each protein assessed by fluorescence or emPAI are taken from Ishihama et al. (2008) and Taniguchi et al. (2010), respectively. All spectra were manually inspected. Phosphopeptides marked with an asterisk were taken from Supplemental Table S1 of Oh et al. (2012).
